# Lysophosphatidylcholine induces cytotoxicity/apoptosis and IL-8 production of human endothelial cells: Related mechanisms

**DOI:** 10.18632/oncotarget.22425

**Published:** 2017-11-10

**Authors:** Mei-Chi Chang, Jang-Jaer Lee, Yi-Jane Chen, Szu-I Lin, Li-Deh Lin, Eric Jein-Wen Liou, Wei-Ling Huang, Chiu-Po Chan, Chi-Chia Huang, Jiiang-Huei Jeng

**Affiliations:** ^1^ Biomedical Science Team, Chang Gung University of Science and Technology, Kwei-Shan, Taoyuan City, Taiwan; ^2^ Department of Dentistry, Chang Gung Memorial Hospital, Taipei, Taiwan; ^3^ School of Dentistry and Department of Dentistry, National Taiwan University Medical College and National Taiwan University Hospital, Taipei, Taiwan; ^4^ Department of Dentistry, Municipal Taoyuan Hospital, Taoyuan City, Taiwan; ^5^ Department of Dentistry, Chang Gung Memorial Hospital, Kaohsiung, Taiwan; ^6^ Department of Dentistry, Cardinal Tien Hospital, New Taipei City, Taiwan

**Keywords:** apoptosis, atherosclerosis, cell cycle, cytotoxicity, endothelial cells

## Abstract

Increased levels of oxidized low-density lipoprotein oxLDL) are shown to elevate the risk of cardiovascular diseases such as atherosclerosis, thrombosis, stroke, and myocardial infarction. This is possibly due to the toxic effects of oxLDLs on vascular cells. Various oxLDLs including lysophosphatidylcholine (LPC) and 7-ketocholesterol injure vascular endothelial cells and stimulate inflammatory reaction. However the toxicity of LPC on endothelial cells is not clear. In this study, human endothelial cells were exposed to LPC. Cytotoxicity was measured by 3-[4,5-dimethylthiazol-2-yl]-2,5-diphenyltetrazolium bromide assay. Propidium iodide (PI) staining or PI/Annexin V dual staining flow cytometry were used to determine cell cycle progression and apoptosis. Reactive oxygen species (ROS) level was analyzed by DCFH-DA labeling flow cytometry. RNA and protein expression of endothelial cells was studied by reverse transcriptase-polymerase chain reaction and western blotting. IL-8 secretion was measured by enzyme-linked immunosorbant assay. LPC showed cytotoxicity to endothelial cells (>50 µg/ml). LPC induced cell cycle arrest and apoptosis with concomitant inhibition of cdc2 and cyclin B1 expression. LPC stimulated intracellular ROS production and ATM/Chk2, ATR/Chk1 and Akt activation. IL-8 expression and secretion in endothelial cells were induced by LPC. LPC-induced apoptosis, and IL-8 expression/secretion was attenuated by LY294002, a PI3K/Akt inhibitor. These results reveal that LPC is involved in the pathogenesis of atherosclerosis and vascular diseases by stimulation of inflammation and injury to endothelial cells. These events are related to ROS, ATM/Chk2, ATR/Chk2 and PI3K/Akt signaling. Understanding the toxic mechanisms of LPC is useful for future prevention and treatment atherosclerosis.

## INTRODUCTION

Cardiovascular diseases (stroke, myocardial infarction etc.) are the major systemic diseases of peoples in the world. This is possibly due to increased levels of oxidized low-density lipoprotein (oxLDL) that elevate the risk of cardiovascular diseases. Oxidized low-density lipoprotein (Ox-LDL) contains mainly lysophosphatidylcholine (LPC), lipid ester-bound aldehydes, 7-ketocholesterol (7-KC), 7α-hydroxycholesterol, 7β-hydroxycholesterol, 5α,6α- epoxycholesterol, 5β,6β-epoxycholesterol, 25-hydroxycholesterol, (25R)-26- hydroxycholesterol), and cholesta-3,5-dien-7-one [1]. These ox-LDLs show differential toxic effects toward vascular smooth muscle cells, endothelial cells and macrophages [2]. LPC is one major oxLDL involved in many diseases. LPC and oxidized non-esterized fatty acids are also generated by lipoprotein-associated phospholipase A2 (Lp-PLA2) and linked to the pathogenesis of atherosclerosis, myocardiac infarction, and stroke. LPC also modulates various disease-related mechanisms and is involved in many diseased processes including diabetes, obesity, atherosclerosis and cancer [3]. LPC has been shown to induce apoptosis of human coronary artery smooth muscle cells via activation of TRPC1/TRPC3 channels, calcium influx, Bax and caspase-3 and contributes the atherosclerosis and coronary artery disease [4]. LPC also has the ability to impair endothelium-dependent vasorelaxation, enhance endothelial proliferation and permeability, induce adhesion and activation of lymphocytes, initiate macrophage chemotaxis and stimulate the activities of vascular smooth muscle cells and platelets [5]. Therefore targeting therapy against Lp-PLA2 and LPC are recently recommended for treatment of associated diseases [6].

Inflammatory cell infiltration of vascular walls, reactive oxygen species (ROS) production, and apoptosis of endothelial cells are involved in the pathogenesis of atherosclerosis. Oxidative stress may stimulate inflammatory response of endothelial cells by inducing the release of various cytokines such as interleukin-1 (IL-1), tumor necrosis factor-α (TNF-α), IL-6, IL-4, chemokines and cell adhesion molecules (e.g., intercellular adhesion molecule-1 [ICAM-1], E-selectin etc.), leading to infiltration of inflammatory cells through endothelial cells into tissue and contributing to atherosclerosis [7]. LPC, as one major ox-LDL, has been shown to induce monocyte chemotactic protein-1 (MCP-1), IL-6 expression and cytotoxicity/apoptosis to endothelial cells via Notch signaling [8]. LPC also induces IL-6 and IL-8 production in endothelial cells [9]. LPC markedly inhibited nitric oxide (NO) production, but increased the levels of ROS, and maelic dialdehyde of endothelial cells [10].

Level of ROS in tumor tissues and atherosclerotic tissues is generally higher than healthy tissues [11]. The sources of ROS in endothelial cells are derived mainly from NADPH oxidase, xanthine oxidase, arachidonic acid (AA) metabolism or mitochondrial electron transfer. LPC has been shown to be the ROS inducer in endothelial cells [12–15] and may induce oxidative DNA damage and gene hypomethylation in cholangiocytes [16]. ROS and DNA damage may stimulate ataxia-telangiectasia mutated (ATM)/checkpoint kinase-1 (Chk2) and ATM and RAD3-related (ATR)/Chk1 to regulate cell cycle progression and induce apoptosis [17–19]. Moreover, ROS may provoke phosphoinositide 3-kinase (PI3K)/protein kinase B (Akt) signaling to mediate inflammatory response [20]. Antioxidants and NADPH oxidase inhibitor - diphenylene iodonium (DPI) effectively suppress the IL-4-induced ROS production, IL-6 and MCP-1 secretion of endothelial cells [7].

We hypothesized that LPC generated by oxidation of LDL or by Lp-PLA2 etc. may contribute to cardiovascular diseases via inducing injury, ROS production and inflammatory mediators’ production in vascular endothelial cells. We therefore designed this study to evaluate the effect of LPC on cytotoxicity, cell cycle progression, apoptosis, ROS and IL-8 production, and related signal transduction pahways (ATM/Chk2, ATR/Chk1, and PI3K/Akt) in vascular endothelial cells.

## RESULTS

### Cytotoxicity of LPC to endothelial cells

**Figure 1:**
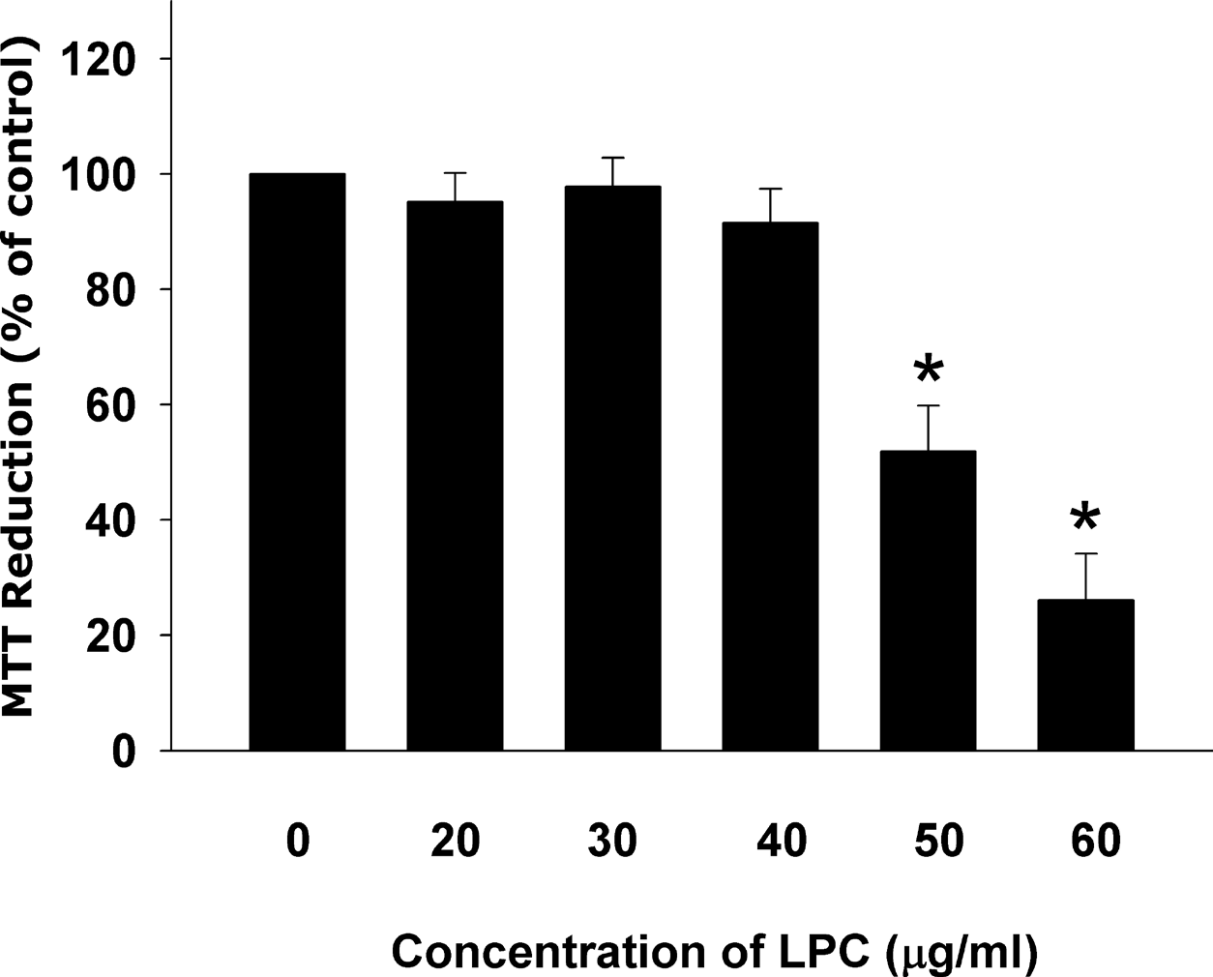
Cytotoxicity of LPC to endothelial cells as analyzed by MTT Results were expressed as Mean ± SE. *denotes statistically significant difference (*p* < 0.05) when compared with solvent control.

Lysophosphatidylcholine (LPC) showed cytotoxicity to Eahy926 (EAHY) endothelial cells. Evident cytotoxicity of LPC was noted at concentrations higher than 50 μg/ml with a fifty percent inhibitory concentration (IC50) of about 50.73 μg/ml (Figure 1).

### Induction of cell cycle arrest and apoptosis of endothelial cells by LPC

**Figure 2:**
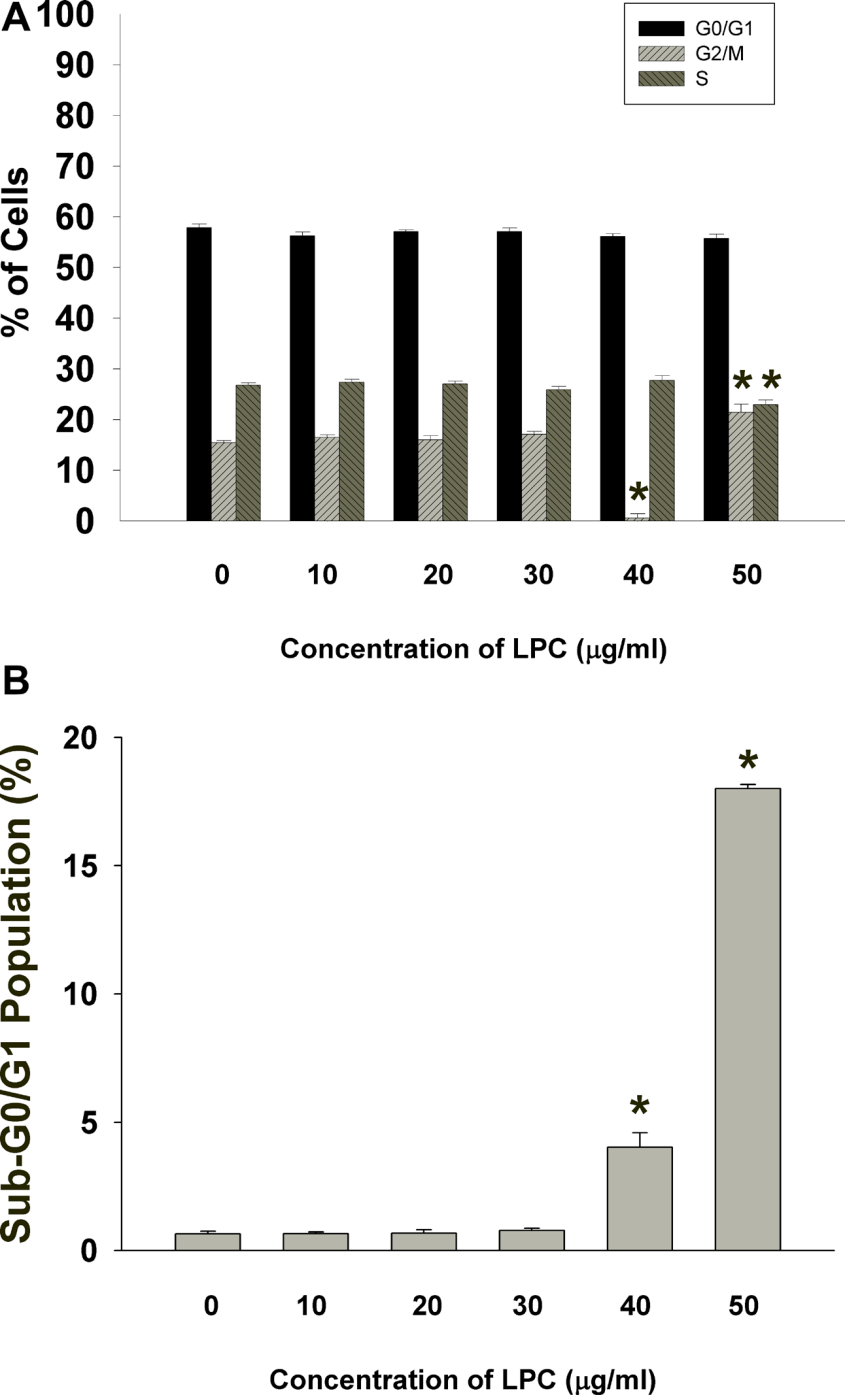
PI fluorescence of EAHY cells after exposure to different concentrations of LPC (10-50 mg/ml) (**A**) EAHY cell populations residing in G0/G1, S, and G2/M after exposure to LPC, (**B**) Sub G0/G1 population of EAHY cells after exposure to LPC. Results were expressed as Mean ± SE. *indicates statistically significant difference when compared with solvent control-treated group.

LPC also induced cell cycle arrest and apoptosis of EAHY endothelial cells. LPC induced G2/M cell cycle arrest of endothelial cells, at a concentration of 50 μg/ml (Figure 2A). The apoptotic population (sub-G0/G1 population) increased by LPC at concentrations of 40 and 50 μg/ml (Figure 2B).

### Induction of apoptosis of endothelial cells by LPC

**Figure 3:**
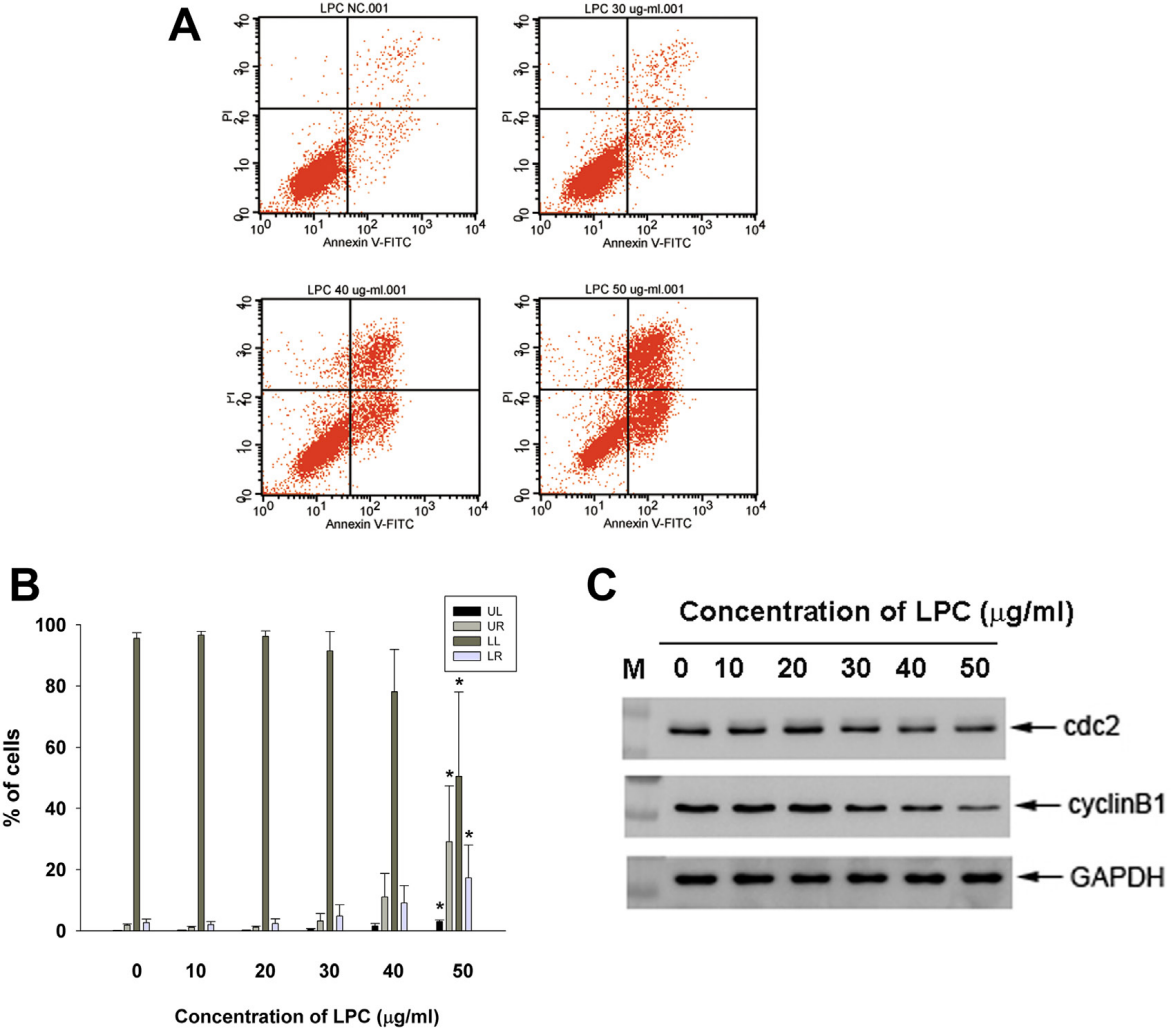
Effect of LPC on apoptosis of EAHY endothelial cells as analyzed by PI and annexin V dual fluorescent flow cytometry (**A**) One representative flow cytometry picture was shown. (**B**) Quantitative analysis of PI + annexin V flow cytometric analysis. *denotes statistically significant difference (*p* < 0.05) when compared with control (**C**) Effect of LPC on cdc2, and cyclin B1 protein expression of EAHY cells. One representative western blot picture was shown. Expression of GAPDH was used as control.

LPC also induced apoptosis of endothelial cells at concentrations higher than 30 μg/ml as analyzed by PI + Annexin V flow cytometric analysis. Increase in upper right (late apoptosis) and lower right (early apoptosis) population of endothelial cells was observed by 50 μg/ml LPC (Figure 3A, 3B).

### Effect of LPC on cell cycle-related protein expression of endothelial cells

Western blotting analysis showed that exposure of EAHY cells to LPC inhibited cdc2 and cyclin B1 protein expression (Figure 3C), a possible reason for induction of G2/M phase arrest and apoptosis.

### Stimulation the p-ATM, p-ATR, p-chk1, and p-chk2 protein expression of EAHY cells by LPC

**Figure 4:**
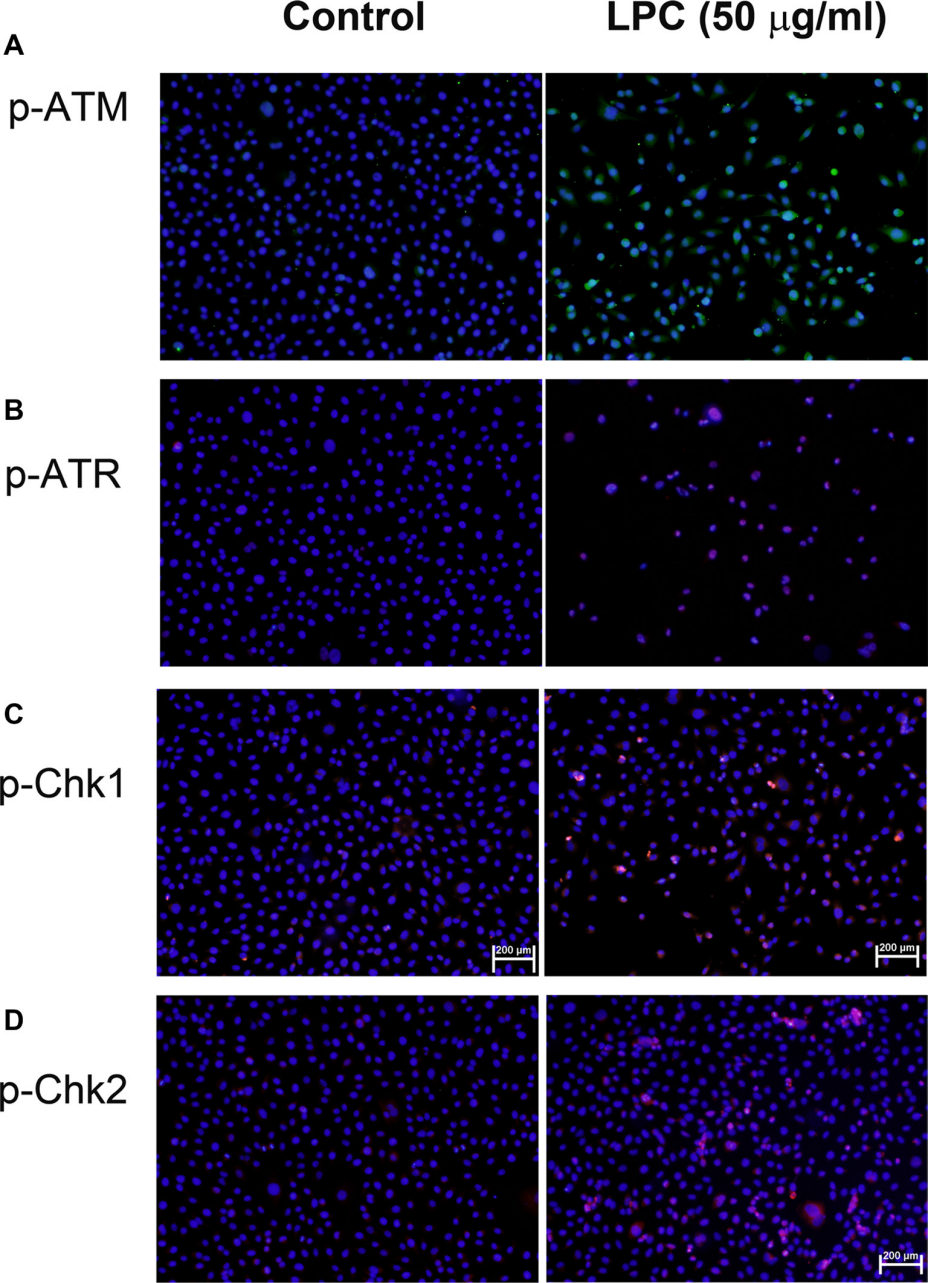
Induction of p-ATM, p-ATR, p-Chk1, and p-Chk2 expression by LPC to endothelial cells EAHY endothelial cells were exposed to different concentrations of LPC. Immunofluorescent (IF) microscopic observation was utilized to determine the expression of (**A**) p-ATM, (**B**) p-ATR, (**C**) p-Chk1, and (**D**) p-Chk2 in endothelial cells. One representative IF picture was shown. (blue – DAPI, green – p-ATM, red – p-ATR, p-Chk1, or p-Chk2).

LPC induced ATM and ATR phosphorylation of endothelial cells as revealed by an increase in FITC (green) and TRITC (red) fluorescence, respectively (Figure 4A, 4B). Similarly, LPC also stimulated p-Chk1 and p-Chk2 phosphorylation of endothelial cells (red fluorescence, Figure 4C, 4D).

### Effect of LPC on IL-8 secretion and expression of endothelial cells

**Figure 5:**
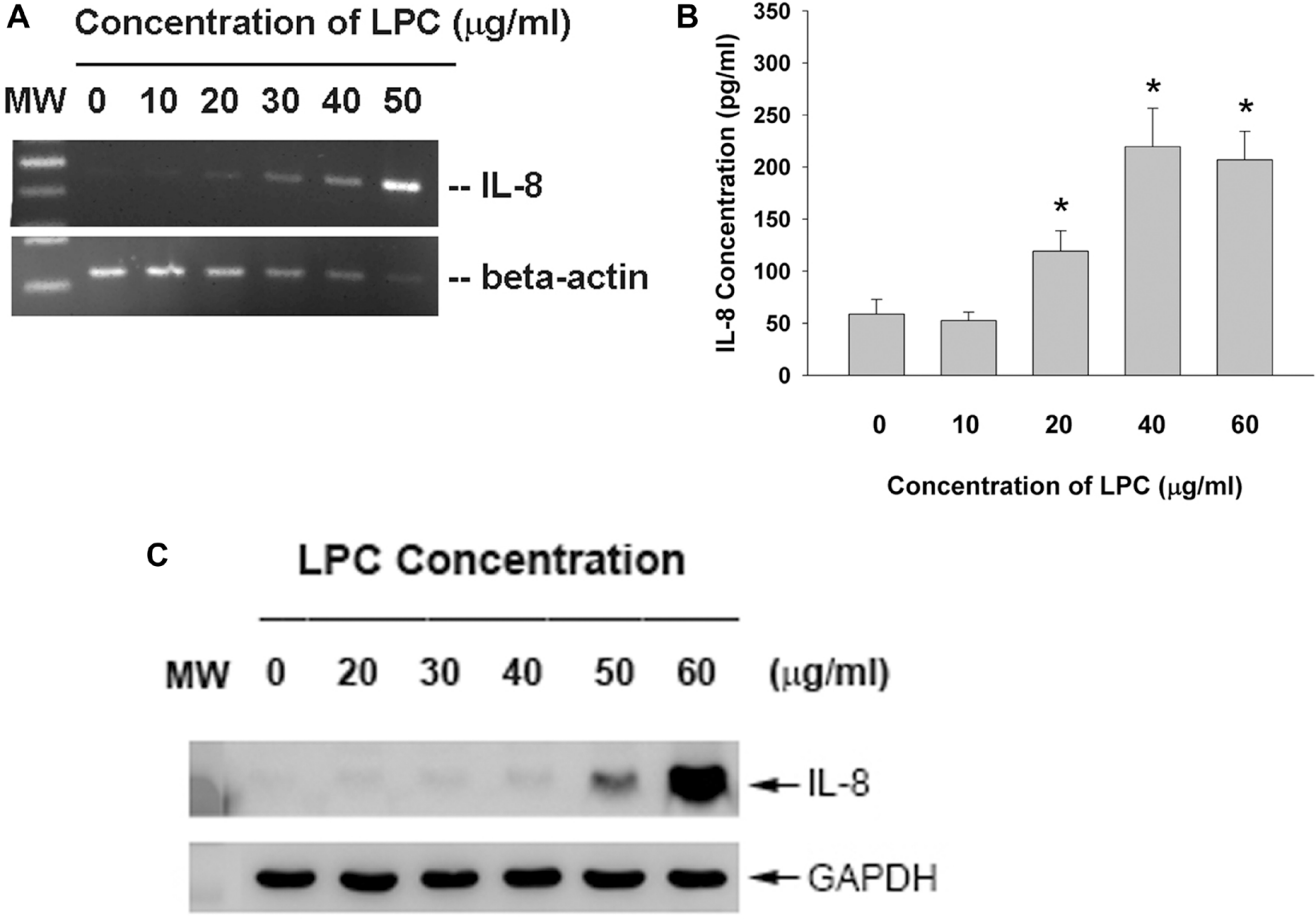
Effect of LPC on IL-8 expression and production EAHY endothelial cells were exposed to LPC. (**A**) Total RNA was isolated and subjected to RT-PCR analysis of IL-8 expression, (**B**) Culture medium was collected and used for measurement of IL-8 secretion in endothelial cells. (**C**) Western blotting of IL-8 protein expression after exposure of endothelial cells to LPC for 24 hours. One representative result was shown.

LPC markedly induced IL-8 expression of endothelial cells (Figure 5A). Moreover, LPC further stimulated the production of IL-8 in endothelial cells as analyzed by ELISA. Concentration of IL-8 in the culture medium was stimulated from 58.8 pg/ml (control) to 119.2, 219.7 and 206.9 pg/ml, by 20, 40 and 60 μg/ml of LPC respectively (Figure 5B). In consistent, LPC also induced IL-8 protein expression of endothelial cells as studied by western blotting (Figure 5C).

### LPC induces ROS production and Akt phosphorylation of endothelial cells

**Figure 6:**
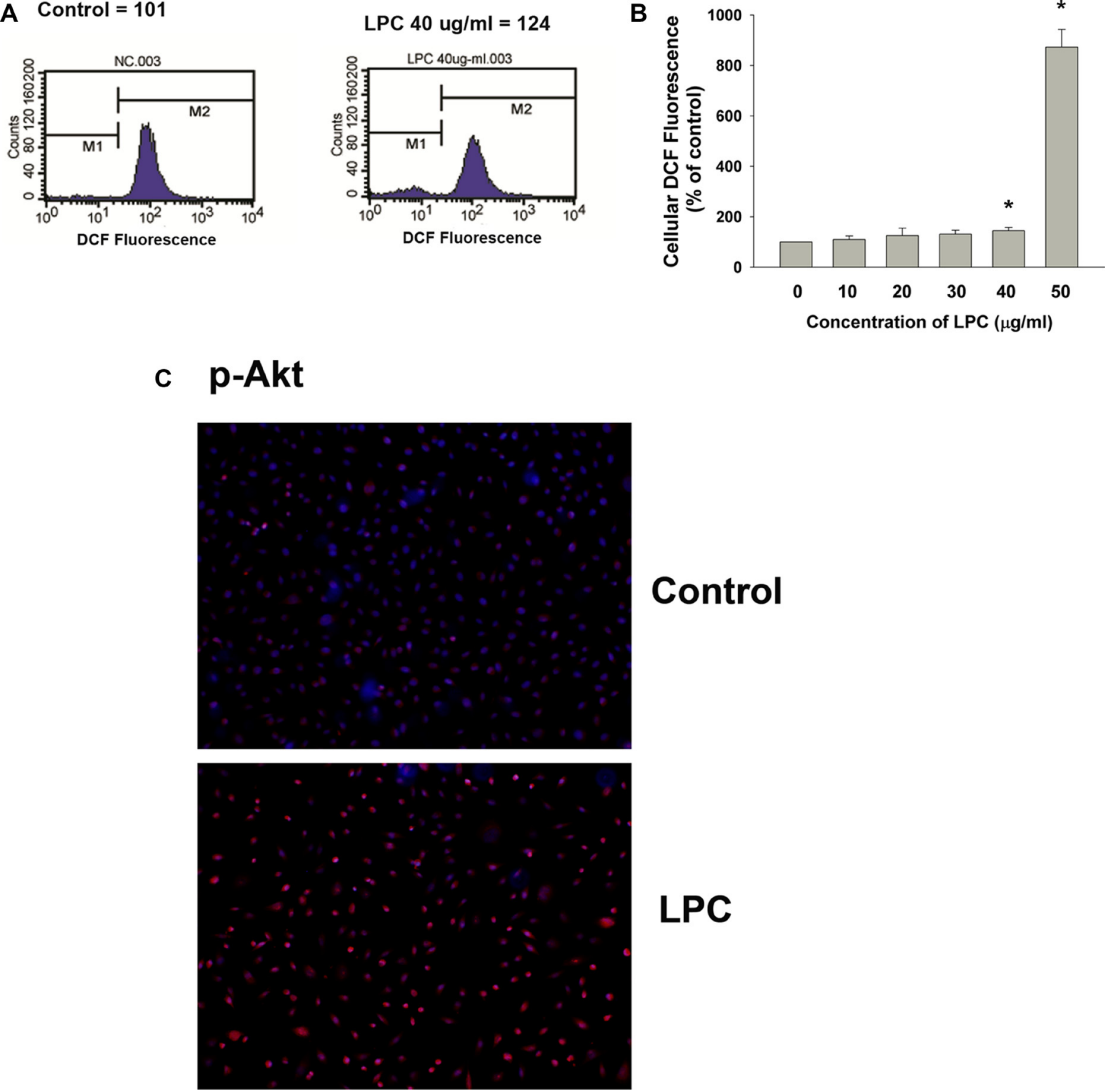
Effects of LPC on cellular ROS levels and Akt phosphorylation (**A**) LPC stimulated ROS production as revealed by an increase in cellular DCF fluorescence. One representative DCF flow cytometry picture was shown, (**B**) Quantitative results of DCF fluorescence. Results were expressed as Mean ± SE (% of control), *denotes statistically significant difference (*p* < 0.05) when compared with control, (**C**) LPC induced Akt activation as indicated by an increase in cellular p-Akt fluorescence (blue – DAPI, red – p-Akt). One representative IF picture was shown.

LPC provoked ROS production of endothelial cells as analyzed by 2’,7’-Dichlorodihydrofluorescin (DCF) fluorescence flow cytometric analysis. Relative DCF fluorescence value of endothelial cells increased from 101 (control) to 124 (40 μg/ml LPC) (Figure 6A). Quantitatively LPC (40–50 μg/ml) stimulated ROS production of endothelial cells with an increase in cellular DCF fluorescence (Figure 6B). Immunofluorescent staining study also revealed the stimulation of Akt phosphorylation of endothelial cells by LPC, as shown by an increase in p-Akt (red) fluorescence (Figure 6C).

### Role of PI3K/Akt signaling on LPC-induced apoptosis and IL-8 expression of endothelial cells

**Figure 7:**
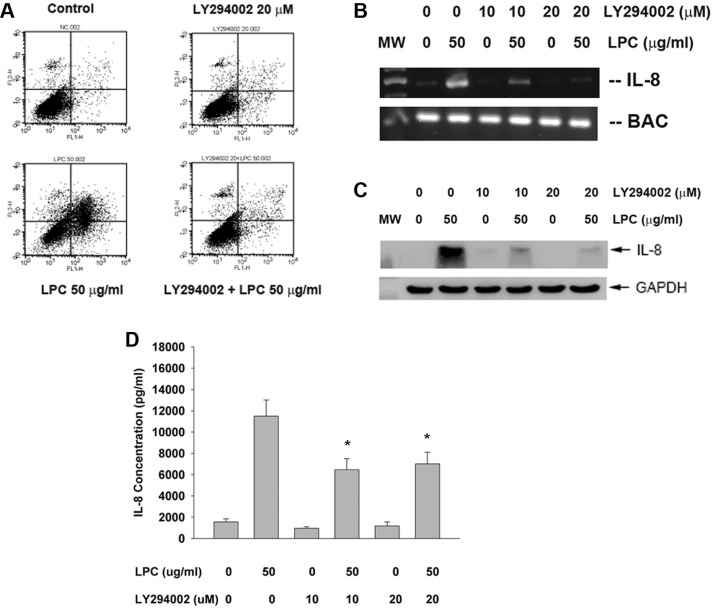
Effect of LY294002 on LPC-induced apoptosis and IL-8 expression/ secretion (**A**) LY294002 prevented the LPC-induced apoptosis of endothelial cells as revealed by PI+Annexin V dual staining flow cytometry. One representative result was shown. (**B**) LY294002 prevented the LPC-induced IL-8 expression of endothelial cells, (**C**) LY294002 prevented the LPC-induced IL-8 protein expression of endothelial cells as analyzed by western blot, (**D**) LY294002 attenuated the LPC-induced IL-8 secretion of endothelial cells as analyzed by ELISA. *denotes statistically significant difference when compared with LPC 50 mg/ml group.

Interestingly, LY294002 (a PI3K/Akt signaling inhibitor) attenuated the LPC-induced apoptosis of endothelial cells as shown by PI and annexin V dual fluorescent flow cytometry (Figure 7A). The percentage of pro-apoptotic cells (LR) and apoptotic (UR) cells decreased obviously after pretreatment and co-incubation of LPC with LY294002. Consistently LY294002 prevented the LPC-stimulated increase of IL-8 mRNA/protein expression and secretion in endothelial cells (Figure 7B, 7C, 7D).

## DISCUSSION

Oxidized low density lipoproteins (oxLDL) such as LPC, 7-KC, 7α-hydroxycholesterol, 7β-hydroxycholesterol, 5α, 6α- epoxycholesterol, etc. may increase the risk of various cardiovascular diseases including atherosclerosis, thrombosis, stroke, myocardial infarction etc [1]. This is possibly due to toxic effects by various ox-LDLs toward vascular smooth muscle cells, endothelial cells and macrophages [2]. LPC may impair endothelium-dependent vasorelaxation, enhance endothelial proliferation and permeability, induce adhesion and activation of lymphocytes, initiate macrophage chemotaxis and modulate the activities of vascular smooth muscle cells and platelets [5]. LPC (5–10 μg/ml) stimulates proliferation, but induces apoptosis (50–300 μg/ml) of endothelial cells via NADPH-dependent superoxide production [14, 15]. In this study, LPC was also shown to suppress endothelial cell proliferation and exhibited cytotoxicity. The dual effects of LPC on proliferation and apoptosis of endothelial cells depend on concentration and exposure time [15]. By toxic damage to endothelial cells, LPC may increase the permeability of endothelium and expose of underlying collagen to promote platelet aggregation/thrombosis.

The toxic effect of LPC is possibly related to its induction of cell cycle arrest and apoptosis of endothelial cells. In this study, LPC obviously induced G2/M and S- cell cycle arrest and apoptosis of endothelial cells. Similarly LPC stimulates apoptosis of HUVEC endothelial cells via NADPH-dependent superoxide production [14, 15] and H19-7 neuroprogenitor cells through the up-regulation of FasL expression and activation of NF-kB [21]. Zhou et al. (2006) shows that LPC induces mainly necrosis of endothelial cells, whereas 7-KC induces endothelial cell apoptosis [22]. These differential results of LPC are possibly related to concentrations, exposure time, cell types etc. In this study, the induction of cell cycle arrest can be partly explained by the decrease of cdc2 and cyclin B1 expression in endothelial cells by LPC. The G2/M phase of cell cycle is critically regulated by cdc2, cyclin B1 and p21 [23, 24]. However, limited information is known about the effect of LPC on cell cycle-related proteins and more studies are needed to clarify these points.

Little is known about the effect of LPC on ATM/Chk2 and ATR/Chk1, two check-point kinase signaling pathways to mediate ROS and DNA damage responses for control of cell cycle progression [17–19]. In this study, we further studied and intriguingly found the activation of p-ATM/p-Chk2 and p-ATR/p-Chk1 of endothelial cells by LPC, implicating the possible ROS overproduction and DNA damage response. Toxic chemicals may induce transcription, cell cycle arrest, apoptosis, cellular senescence and DNA repair via activation of ATM/Chk2 and ATR/Chk1 signaling [17], thereby inhibit cdc2 and cyclin B1 expression via activation of p53 and cdc25C [25]. This may lead to G2/M cell cycle arrest and apoptosis of cells [25] and could partly explain the LPC-induced cell cycle arrest, apoptosis and the suppression of cdc2 and cyclin B1 expression in endothelial cells in this study.

The above event is related to the induction of ROS over-production by LPC. Recently oleoyl-LPC has been shown to cause a decrease of NO production, and eNOS uncoupling, but increase of ROS production in EAHY endothelial cells [13]. Peng et al. (2010) also found the inhibition of NO, stimulation of ROS by LPC to stimulate caspase 3-dependent apoptotic response in endothelial cells [10]. Similarly we also found the over-production of ROS of endothelial cells by LPC, suggesting its involvement in LPC-induced toxic events. Accordingly LPC is shown to stimulate over-production of NO and ROS to mediate its toxic injury to endothelial cells (HUVEC) [12]. LPC stimulates superoxide production of HUVEC endothelial cells through activation of NADPH oxidase to stimulate cell proliferation [14, 15].

Vascular inflammation is important in the pathogenesis of atherosclerosis and other cardiovascular diseases [26, 27]. Local production of chemokines such as IL-8 and MCP-1 may lead to inflammatory cells infiltration in the vascular intima that is important to the initiation and progression of atherosclerosis, heart disease and stroke [28]. In this study, LPC induced IL-8 production and mRNA/protein expression in EAHY endothelial cells. Similarly, LPC is also shown to stimulate IL-6 and IL-8 production of umbilical vein endothelial cells via a sterol regulatory element binding protein-2-independent manner [9]. This is partly due to activation of G-protein coupled receptor, but not platelet activating factor receptor [29]. LPC-induced tissue inflammation (MCP-1, IL-6 secretion) and cytotoxicity to HUVEC is related to notch signaling and g-secretase activity [8]. All these results suggest the involvement of LPC-induced vascular inflammation in vascular diseases.

LPC has been shown to stimulate Akt signaling pathway to up-regulate the production of extracellular matrix proteins such as biglycan, and type I collagen in human aortic valve cells [30]. But this effect of LPC is not mediated by MEK/ERK signaling [30]. These effects by LPC may contribute to valve sclerosis as well as aortic stenosis [30]. Limited information is known about the effect of LPC on PI3K/Akt signaling of endothelial cells. LPC is shown to stimulate Sp1 binding and endothelial nitric oxide synthase (eNOS) promoter activity in endothelial cells via G-protein and PI3K-JAK2-MEK-ERK signaling pathways, but unrelated to Ras and Raf [31]. LPC may stimulate MEK/ERK, Akt and p38 in endothelial cells, but it also inhibits EGF-induced activation of Akt [29, 32]. In this study, we found that LPC induced Akt phosphorylation/activation of endothelial cells. Moreover, LY294002 prevented the LPC-induced apoptosis and IL-8 production/expression of endothelial cells, implicating the crucial role of PI3K/Akt signaling in LPC-induced apoptotic and inflammatory response in vascular endothelium.

In conclusion, these results indicate that LPC may contribute to the pathogenesis of atherosclerosis and other cardiovascular diseases by inducing the cytotoxicity, cell cycle arrest and apoptosis of endothelial cells. These events are related to ROS production, activation of ATM/Chk2, ATR/Chk1, PI3K/Akt and the inhibition of cdc2 and cyclin B1 expression. LPC may also induce vascular inflammation by stimulation of IL-8 production and expression of endothelial cells through activation of PI3K/Akt signaling. These results can be helpful for our understanding the physiological and toxicological effect of LPC on the health of cardiovascular system, the underlying signal transduction mechanisms and future disease prevention.

## MATERIALS AND METHODS

### Materials

3-(4,5-dimethylthiazol-2-yl)-2,5-diphenyl tetrazolium bromide (MTT), DCFH-DA and dimethylsulfoxide (DMSO) were purchased from Sigma/Aldrich Chemical Company (St. Louis, MO, USA). LPC and LY294002 were purchased from Cayman (Cayman Chemical Company, Ann Arbor, MI, USA). Cell culture biologicals (Dulbecco’s modified Eagle’s medium [DMEM], fetal bovine serum [FBS], trypsin/EDTA etc) were obtained from Life Technologies (Grand Island, New York, USA). Eahy926 (EAHY) endothelial cells were given by Professor Cora-Jean S. Edgell (North Carolina University, NC, USA) and characterized to express endothelial cell markers such as factor VIII-related antigens and von Willebrand factor [33] and studied in my laboratory before [34, 35]. They were cultured in DMEM containing 10% FBS. Specific PCR primers were synthesized by MDBio Inc. (Taipei, Taiwan). Antibodies against cdc2 (sc-54), cyclin B1 (sc-245), p-ATM (Ser1981, sc-47739), p-ATR (Ser428, sc-109912), p-Chk1 (Ser345, sc-17922), p-Chk2 (Thr68, sc-16297-R), p-Akt 1/2/3 (Ser473, sc-514032) and glyceraldehtde 3-phosphate dehydrogenase (GAPDH) (sc-32233) were obtained from Santa Cruz (Santa Cruz Biotechnology, Dallas, TX, USA). Enzyme-linked immunosorbant assay (ELISA) kits for IL-8 were purchased from PeproTech (Rocky Hill, NJ, USA). RT and PCR kits were from Zimeset Biotech Co. Ltd, Taipei, Taiwan. Annexin V-FITC and isotype control were from eBioscience (San Diego, CA, USA).

### Cytotoxicity of LPC on endothelial cells

Briefly, 5 × 10^5^ EAHY cells were seeded onto 6-well culture plates. After 24 h, culture medium was changed and then various amounts of LPC (10-50 μg/ml) were added. Cells are further incubated for 3 days. Culture medium was collected for ELISA. Then fresh medium containing MTT (final 0.5 mg/ml) was added into each well and cells were cultured for further 2 h. The insoluble formazan generated by viable cells was dissolved in DMSO and read against reagent blank (DMSO) at a wavelength of 540 nm by a microplate reader (BioTech Instruments Inc., VM, USA) as before for estimation of cell viability [36–38]. Results were expressed as Mean ± SE (% of control).

### Effect of LPC on cell cycle progression

Briefly 5 × 10^5^ EAHY were seeded onto 6-well culture plates. After 24 h, culture medium was changed and then various amounts of LPC (final 10, 20, 30, 40, 50 μg/ml) were added. Cells were further incubated for 3 days. Alterations in the cell cycle distribution of endothelial cells were investigated by propidium iodide (PI) staining of DNA contents by flow cytometry [36–38]. Briefly, both floating and attached cells were collected together, re-suspended and fixed for 30 min in 70% ice-cold ethanol including RNase (2 mg/ml). Cells were then washed with phosphate-buffered saline (PBS) and finally stained with PI (40 µg/ml) for 10 min. The PI-elicited fluorescence of individual cell was measured by a FACSCalibur Flow Cytometer (Becton Dickinson, Worldwide Inc., San-Jose, CA, USA). The wavelength of laser excitation was set at 488 nm and the emission collected was set at greater than 590 nm. The FL2 fluorescence was collected in a linear/log scale fashion. A total of 10,000 cells were analyzed for each sample. The percentage of cells residing in G_0_/G_1_ phase, S phase, G_2_/M and sub-G_0_/G_1_ phase were measured using standard ModiFit software and CELL QUEST programs.

### Assay the effect of LPC on apoptosis of endothelial cells - PI+Annexin V dual fluorescent flow cytometry

Endothelial cells (5 × 10^5^ cells) were seeded and treated by various concentration of LPC for 24 hours. Then both floating and attached cells were harvested. Cells were then washed with PBS, resuspended in 400 μl HEPES (10 mM HEPES-NaOH, pH 7.4, 140 mM NaCl, 2.5 mM CaCl_2_) solution, and the Annexin V-FITC (Becton Dickson)/ PI (50 μg/ml) staining solution was added in the dark for 30 min. The Annexin V-FITC and PI fluorescence of cultured cells were analyzed by FACSCalibur Flow Cytometry (Becton Dickinson) immediately as described before [36]. In each analysis, 15,000 events will be recorded.

### Reverse transcriptase-polymerase chain reaction (RT-PCR)

Generally 1.5 × 10^6^ EAHY cells were inoculated onto 10-cm culture dishes and exposed to LPC for 24h. Total RNA was isolated and subjected for analysis of IL-8 and beta-actin genes expression by RT-PCR procedures as before [36, 37]. In brief, RNA was reverse transcribed for generation of cDNA and the same amounts of cDNA were used for PCR amplification in a reaction mixture composing 5 μl of 10x Super TAQ buffer, 4 μl of 2.5 mM dNTP, 1 μl of each specific primer, 0.2 ml of Super TAQ enzyme (2 U/μl), and double distilled water. The nucleotide sequence of PCR primers were BAC: AAGAGAGGCATCCTCACCCT and TACATGGCTGGGGTGTTGAA (218 bp), and IL-8: CACAAGAGCCAGGAA GAAAC and CACAAGAGCCAGGAAGAAAC (459 bp) [35]. The amplification steps for the studied genes included 20-35 cycles of PCR, denaturing at 94°C for 1 min, annealing at 55°C for 1 min, and extension at 72°C for 1 min. This was followed by a final extension at 72°C for 7 min. The PCR amplified products were loaded into 1.8% agarose gel electrophoresis, and DNA bands were stained with ethidium bromide for photograph taking.

### Western blotting

Generally 1.5 × 10^6^ EAHY were inoculated onto 10-cm culture dishes and exposed to various concentrations of LPC for 24h. After removal of medium and washed with PBS, cell lysates were prepared by dissolving cells in lysis buffer (10 mM Tris-HCl, pH 7; 140 mM sodium chloride; 3 mM magnesium chloride; 0.5% NP-40; 2 mM phenylmethylsulfonyl fluoride; 1% aprotinin; and 5 mM dithiothreitol). Then the equal amounts of proteins (20–50 μg/ml) were loaded to run 12.5% sodium dodecyl sulfate-polyacrylamide gel electrophoresis (SDS-PAGE) for protein separation and transferred to a polyvinylidene fluoride (PVDF) membrane. The membranes were first blotted with primary antibodies of cdc2, cyclin B1, IL-8 and GAPDH for 2 hr as described before [35, 36]. The membranes were then incubated in respective horseradish peroxidase-link secondary antibodies (Jackson ImmunoResearch Laboratories, West Grove, PA, USA) for 1 hr. After washing the membrane with buffer, ECL reagents (Amersham) were added and the chemiluminescence of protein bands was determined by exposure of membranes to Fuji films for 30 sec to 10 min. The intensity of GAPDH bands was used as control.

### Effect of LPC on IL-8 production of endothelial cells as analyzed by ELISA

In short, 5 × 10^5^ EAHY cells were inoculated onto 6-well culture plates. After 24 h, culture medium was changed with fresh medium containing various amounts of LPC (final concentrations of 10, 20, 30, 40, 50 mg/ml) for 3 days. Culture medium was collected for analysis of IL-8 by ELISA following the instruction of assay kits [35, 37, 39, 40].

### Effect of LPC on cellular ROS levels

Briefly 5 × 10^5^ EAHY were seeded onto 6-well culture plates. After 24 h, culture medium was changed and then various amounts of LPC (final 10, 20, 30, 40, 50 mg/ml) were added. Cells were further incubated for 3 days and then stained with 10 μM DCFH-DA for 30 minutes. Both floating and attached cells were collected and pulled together. Cells were re-suspended in 200 μl of PBS and the DCF-elicited fluorescence of cells was determined by FACS Calibur Flow Cytometry (Becton Dickinson, Worldwide Inc., San-Jose, CA, USA) [35, 36]. Totally 10000 cells were analyzed for all the samples. The mean of DCF fluorescence was counted by the CELL Quest program (Becton Dickinson, CA, USA).

### Activation of ATM, ATR, chk1, chk2 and akt by LPC

EAHY cells (1 × 10^5^) were seeded onto 24-well culture plate with sterile coverslips in 1 ml DMEM with 10% FBS. After 24 h, culture medium was changed and then various amounts of LPC (final 10, 20, 30, 40, 50 μg/ml) were added. Cells were further incubated for 24 hours or different time points. Then medium was removed, and cells were washed with PBS and fixed in 4% paraformaldehyde for 20 minutes. Cells were further washed with PBS, permeabilized with 2% Triton X-100, exposed to 0.3% v/v H_2_O_2_ for 20 minutes. After rinsed with PBS, 5% bovine serum albumin (BSA) was used for blocking cells for 1 hour and then cells were incubated with primary antibodies (p-ATM, p-ATR, p-Chk1, p-Chk2, p-Akt) (1:1000, v/v) at room temperature for overnight. Following washed by PBS, cells were incubated in respective secondary antibodies (FITC-conjugated for p-ATM, and TRITC-conjugated for p-ATR, p-Chk1, p-Chk2 and p-Akt) in the dark for 1 hour and counterstained the nucleus with DAPI (1:1000) for 30 min. Finally the cells in coverslips were mounted and photographed/observed under an inverted microscope and DP Controller/Manager software (Olympus IX71, Olympus Corporation) [35, 36, 38].

### Role of PI3K/Akt signal transduction pathway in LPC-induced changes of endothelial cells

To know the role of PI3K/Akt signaling, EAHY endothelial cells (5 × 10^5^ cells/well, in 6-well plate) were pretreated by LY294002 (10/20 μM, a PI3K/Akt signaling inhibitor) or DMSO for 30 min prior the addition of LPC, and cells were further co-incubated for 3 days. Cellular apoptosis was measured by PI/Annexin V dual fluorescent flow cytometry as described above. IL-8 mRNA expression, protein expression and secretion were studied by RT-PCR, western blotting and ELISA, respectively, as described above.

### Statistical analysis

Three or more separate experiments were conducted. The results were analyzed by paired Students *t*-test. A *p* value < 0.05 was considered to have a statistically significant difference between groups. In some experiments, 50% inhibitory concentration (IC50) of LPC was calculated by Regression analysis.
